# The SOFT Cluster Score as a Multifaceted Predictive Model for Postoperative Outcomes

**DOI:** 10.1097/TXD.0000000000001951

**Published:** 2026-05-06

**Authors:** Gwendolyn B. Henry, Spoorthi Kamepalli, Carter J. Burns, Jeremy Birkmire, George Cholankeril, Avegail Flores, John A. Goss, Abbas Rana

**Affiliations:** 1 Department of Student Affairs, Baylor College of Medicine, Houston, TX.; 2 Division of Abdominal Transplant, Michael E DeBakey Department of Surgery, Baylor College of Medicine, Houston, TX.

## Abstract

**Background.:**

Consideration of both donor and recipient factors at the time of an organ offer is crucial in achieving the most optimal outcome given the shortage of available donor organs and waitlist mortality risk. Our aim was to achieve a comprehensive risk assessment for liver transplant patients by creating a series of independent risk index equations for 6 distinct posttransplant endpoints in the transplant continuum of care.

**Methods.:**

The Survival Outcomes Following Liver Transplantation (SOFT) Cluster score includes risk indices for patient mortality at 90 d, 1 y, 3 y, and 5 y posttransplant, graft failure at 1 y after transplantation, and prolonged length of stay (>30 d). Using a total sample of 61 231 patients, significant risk and protective factors were identified through univariable and multivariable analysis from a training set of 40 821 patients from the Organ Procurement and Transplantation Network database.

**Results.:**

When applied to a validation set of 20 410 patients, the SOFT Cluster score has a *C*-statistic of 0.66 for 90-d mortality, 0.64 for 1-y mortality, 0.63 for 3-y mortality, 0.63 for 5-y mortality, 0.76 for prolonged length of stay, and 0.60 for 1-y graft failure.

**Conclusions.:**

The SOFT Cluster score represents a novel proof-of-concept model for posttransplant risk assessment and provides physicians with a more holistic understanding of patient risk by examining a spectrum of outcomes after liver transplantation up to 5 y posttransplant using a modern cohort of recipients.

## Graphical abstract

**Figure:**
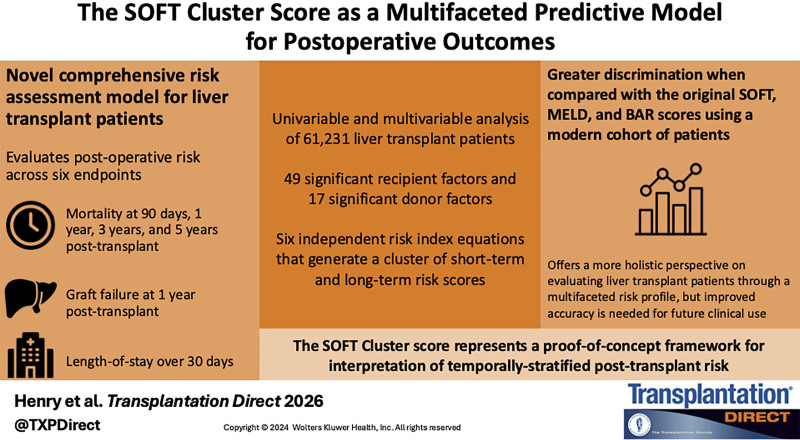

## INTRODUCTION

Liver transplantation is a life-saving surgical procedure for patients with end-stage liver disease. Over the last several decades, there have been marked improvements in posttransplant outcomes that can be attributed to standardization of postoperative management, immunosuppression, and novel intraoperative techniques. However, determining the best candidates for transplantation is still largely left up to the clinical judgment of transplant professionals and therefore represents an opportunity for systematic improvement.^1^ Given the scarcity of viable donor organs, identifying which candidates are most likely to benefit from liver transplantation is crucial to optimize patient outcomes.

Several risk scores have been established to address this problem. The Model for End-Stage Liver Disease (MELD) score, currently used for graft allocation in the United States, accurately predicts 90-d waitlist mortality; however, the MELD score is a poor predictor of posttransplant mortality.^2,3^ The Survival Outcomes Following Liver Transplantation (SOFT) score can accurately predict 90-d posttransplant mortality,^4^ and the balance of risk (BAR) score has been shown to accurately stratify posttransplant survival and uses fewer donor and recipient factors than the SOFT score.^5^ However, these indices reflect only a single endpoint to characterize posttransplant outcomes. In a recent analysis, Shaffer et al^6^ evaluated 7 predictive models with a modern cohort of patients and found that these models are similar with suboptimal calibration at 3- and 5-y survival endpoints. Furthermore, none incorporate the risk of prolonged length of stay (LOS) after liver transplantation.^6^ Understanding a patient’s odds of mortality at a single point in time offers some insight into their survival benefit, but risk scores in isolation do not illustrate a comprehensive picture of patient risk.

The primary hypothesis for this study is that risk factors for short-term and long-term survival are distinct, and our aim is to create specific risk index scores that reflect these differences in which factors are predictive in the short- and long-term. The SOFT Cluster score is a proof-of-concept model that expands upon previous scores to create a more holistic profile of patient risk by evaluating a broader spectrum of posttransplant outcomes. The Cluster score yields 6 unique predictive risk scores for 6 different measurements of patient outcomes. This creates a comprehensive profile of short-term and long-term metrics and offers a better understanding of transplant benefit than an isolated risk score for postoperative mortality at a single point in time. Taking a more holistic approach to understand patient risk is a novel method of analyzing which candidates are best equipped to maximally benefit from liver transplantation and to identify heterogeneity of risk. The SOFT Cluster score represents a novel conceptual framework for interpretation of temporally stratified risk upon which future risk scores can be built for use at the time of transplant. Future iterations of this model can be used to improve risk discussion with prospective recipients and their caregivers as well as improve their perioperative and postoperative management.

## MATERIALS AND METHODS

### Design

This study was a retrospective analysis of liver transplant patients. Institutional Review Board approval was obtained before data analysis, and the requirement for informed consent was waived. The sample used in this study consists of de-identified patient-level data from the Organ Procurement and Transplantation Network (OPTN), which includes all patients listed for liver transplantation in the United States.

All transplant recipients 18 y and older listed in the OPTN liver registry who underwent liver transplantation between April 1, 2014, and July 1, 2023, were included. A total of 61 231 recipients were followed from the date of transplant until death or the date of last known follow-up. Recipients of live-donor transplants (n = 9156) and multivisceral transplants (n = 16 537) were excluded. Demographic characteristics are listed in Table 1.

**TABLE 1.** Demographic characteristics of recipients and donors

Characteristics	Recipient	Donor
Age (y)	55.2 ± 11.4	42.0 ± 16.0
% Female	34.7%	39.1%
% African American	7.6%	18.2%
BMI	29.0 ± 6.0	28.3 ± 6.7
INR	2.0 ± 1.3	N/A
Creatinine (mg/dL)	1.4 ± 1.1	1.8 ± 2.0
MELD	23.8 ± 11.0	N/A
Cold ischemia time (h)	N/A	6.11 ± 2.4
Cause of liver failure		
Noncholestatic cirrhosis	41.40%	N/A
Cholestatic cirrhosis/MASLD	21.82%	N/A
Biliary atresia	0.26%	N/A
Acute hepatic necrosis	4.12%	N/A
Metabolic disease	2.31%	N/A
Malignant neoplasms	22.62%	N/A
Other	4.06%	N/A
Cause of death		
Anoxia	N/A	42.36%
CVA	N/A	28.50%
Head trauma	N/A	26.73%
CNS tumor	N/A	0.34%
Other	N/A	2.07%

BMI, body mass index; CNS, central nervous system; CVA, cerebrovascular accident; INR, international normalized ratio; MASLD, metabolic dysfunction-associated steatotic liver disease; MELD, Model for End-Stage Liver Disease; N/A, not applicable to this group of patients.

### Data Collection

Data were collected by the Organ Transplant and Procurement Network (OPTN).^7^ This analysis used donor and recipient characteristics reported at the time of transplant. Follow-up information was collected at 6 mo posttransplant and annually following transplantation.

### Data Analysis

Statistical analysis was performed using Stata, Version 18.0.^8^ Continuous variables were reported as a mean (SD) values and compared using a Student *t* test. Contingency table analysis was used to compare categorical variables. Results were considered significant at *P* < 0.05, and all reported *P*-values were 2-sided. Using a random split, patients were divided into a training set (n = 40 821) and validation set (n = 20 410) for analysis. Significant recipient and donor risk factors were used to generate odds ratios (ORs) for the 6 primary outcomes, which were then used to create a risk index equation for the Cluster score.

The primary outcomes measured were posttransplant death, graft failure, and LOS. Time to death was measured as the time from transplant date to date of death. Graft failure was defined by the OPTN database and measured as the time from date of transplant to the date of determined graft failure. LOS was measured as date of transplant to date of discharge. Time-to-event analysis was performed using Kaplan-Meier analysis with log-rank test and logistic regression analysis. Ninety-day, 1-y, 3-y, and 5-y survival were the dependent variables for measuring patient survival. One-year graft survival was the dependent variable for measuring graft survival. Hospitalization >30 d was the dependent variable for measuring LOS. Risk factors were the independent variables in the logistic regression analyses for patient survival, graft survival, and LOS. Continuous variables were categorized using clinically relevant groupings. Patients with malignancy do not reflect patients whose cancer was discovered incidentally during transplantation, only those with a known cancer diagnosis before their transplant. Patient functional status is based on their Karnofsky performance score as listed in the OPTN database.

Logistic regression analysis determined the predictors of patient death at 90 d, 1 y, 3 y, and 5 y posttransplant. Logistic regression was used for analysis because of its focus on binary outcomes for a given risk factor at an independent point in time. Donor and recipient variables were first analyzed through univariable logistic analysis, and significant variables were then analyzed through multivariable logistic analysis (**Tables S2–S7**, **SDC**, https://links.lww.com/TXD/A861). Those which demonstrated independent statistical significance through multivariable regression were incorporated into the risk index equations developed for each of the 6 primary outcomes. Every patient starts with a risk score of 1 before factors are incorporated. Each risk equation yields an OR and 95% confidence interval (CI) using the natural log of the OR calculated for each independently significant risk factor. The sum of these scores generates the overall risk score for each patient as shown in Table 2 where the points allotted toward the overall risk score for a given variable A is represented by the OR for that variable multiplied by the binary (0 or 1) outcome of whether the patient in question exhibits that factor A:

**TABLE 2.** Risk index equations and *C*-statistics

Risk factor	Points allotted toward risk score					
	90-d mortality score	1-y mortality score	3-y mortality score	5-y mortality score	Prolonged LOS	1-y graft failure score
*C*-statistic—training set	0.6733	0.6530	0.6502	0.6426	0.7546	0.6343
*C*-statistic—validation set	0.6598	0.6360	0.6278	0.6299	0.7557	0.6031
Recipient						
Age 18–30		–0.4101	–0.2640	–0.2747		
Age 60–65			0.3582	0.3654		
Age > 65	0.4246	0.4967	0.5260	0.4720	0.3522	0.3402
Albumin 2.0–2.5 g/dL	0.1577	0.1455	0.1537			
Ascites					0.1629	
BMI 35–40	0.2824	0.1704	0.1448			0.1581
BMI >40	0.3456					0.1931
Positive hepatitis C serology				0.1263		
Dialysis before transplant	0.3394	0.2844	0.2643	0.1976	0.5008	0.2762
Encephalopathy at transplant	0.1434	0.1947	0.1538	0.1261	0.3880	0.1145
Education level—high school		0.0827	0.1181	0.0988		
Education level—technical school	–0.1514					
African American	0.1911	0.1656	0.3323	0.2695		0.2346
Hepatocellular carcinoma		0.1586	0.2305	0.2339		0.1183
MELD 30–35					0.1874	
MELD 35–40					0.2385	–0.2288
MELD >40					0.3763	0.1816
Serum sodium 130–135 mEq/L					–0.1213	
Serum sodium 145–150 mEq/L	0.4622	0.3747	0.3431	0.3003		0.3089
Serum sodium 150–155 mEq/L	0.8673	0.5852	0.5426	0.4382		0.4123
Serum sodium 155–160 mEq/L	1.5777	1.4061	1.2980	1.1022		1.0475
Moribund functional status (2010)	0.6103	0.3825	0.3823	0.3414		0.4165
Very sick functional status (2020)	0.3820	0.2409	0.2332	0.2226	0.1824	0.2846
Requiring occasional assistance (2060)		–0.2460	–0.2224	–0.1877	–0.2955	–0.1929
Caring for self; unable to carry out normal activity (2070)		–0.2229	–0.1678	–0.1706	–0.5462	–0.1934
Normal activity with effort (2080)		–0.3235	–0.2738	–0.2354	–0.5580	–0.2348
Normal activity with minor symptoms (2090)	–0.8897	–0.5574	–0.4445	–0.3927	–0.4143	–0.3081
INR 2.0–2.5			–0.1155	–0.0892		
INR 3.5–4.0					–0.2398	
INR > 4.0					–0.2158	
ICU pretransplant					0.6678	0.1637
Hospital admission pretransplant					0.3951	
One previous transplant	0.9757		0.7177			
Two previous transplants	1.4362	1.4790	1.3138	1.2332	0.9921	1.4816
Ventilator-dependent pretransplant		0.2694	0.2455	0.2751	0.7471	0.2824
Portal vein thrombosis at transplant	0.3633	0.2890	0.2517	0.2684	0.2207	0.3069
Private insurance			–0.1144	–0.1312	–0.1855	
Previous abdominal surgery	0.2770	0.1580	0.1195	0.1272	0.0913	0.1378
OPTN region 1—CT, ME, MA, NH, RI, East VT		0.2101				0.2029
OPTN region 2—DE, DC, MD, NJ, PA, WV, North VA		0.2459	0.2332	0.2074	0.3096	0.1990
OPTN region 3—AL, AR, FL, GA, LA, MS, PR					–0.1550	
OPTN region 5—AZ, CA, NV, NM, UT				–0.1793		
OPTN region 6—AK, HI, ID, MT, OR, WA				–0.2289		
OPTN region 9—NY, West VT					0.3795	
OPTN region 11—KY, NC, SC, OH, TN, VA					–0.2272	
Total bilirubin <2.0 mg/dL	0.1656		0.1904	0.2284		
Total bilirubin >32.0 mg/dL					0.1918	
TIPS at transplant		0.1564	0.1744	0.1485	0.1666	0.1508
Working for income		–0.2262	–0.2236	–0.2286	–0.4344	–0.1388
Donor						
Age 20–30					–0.1235	–0.1270
Age 65–70			0.1577	0.1263		0.2328
Age >70			0.2063	0.2213		0.4353
Cold ischemia time 0–6 h	–0.1836	–0.1841	–0.1450	–0.1881	–0.1965	–0.2307
Cold ischemia time 12–14 h	0.5440					
Cold ischemia time >14 h			–0.4008	–0.5434		
Controlled donation						0.5165
Creatinine 1.5–2.0 mg/dL					0.2006	
Distance from donor hospital 500–1000 miles					0.1950	
African American						0.1393
Positive hepatitis C serology	–0.2241					
Blood pH < 7.0	1.7372	1.5936				
Regional allocation			–0.0772	–0.1065		
National allocation			–0.1671	–0.3924		
Total bilirubin 1.0–1.8 mg/dL					0.1149	
Weight difference at transplant 45–70 kg				0.1475		
Weight difference at transplant –45 to –70 kg	0.4289					

BMI, body mass index; ICU, intensive care unit; INR, international normalized ratio; LOS, length of stay; MELD, Model for End-Stage Liver Disease; OPTN, Organ Procurement and Transplantation Network; TIPS, transjugular intrahepatic portosystemic shunt.


$$\begin{array}{cc} & risk\;index=\left(ln\left(Odds\;ratio\;for\;variable\;A|A\right)\times variable\;A\right)\\ & +\left(ln\left(Odds\;ratio\;for\;variable\;B|B\right)\times variable\;B)+\ldots \right) \end{array}$$

Receiver operating characteristic (ROC) curve analysis was used to measure the validity of each risk equation. Patients were then split into the 10, 50, and 90 percentiles to create Kaplan-Meier curves for each risk score.

The SOFT Cluster score was compared with 3 currently used systems for transplant risk stratification—the BAR score, original SOFT score, and MELD score. Area under the curves (AUCs) and *C*-statistics were calculated for each scoring system (Table 3), and a DeLong test for discrimination was performed for comparison (**Table S8**, **SDC**, https://links.lww.com/TXD/A861). Additionally, Cox proportional hazards models were fit for further assessment of long-term outcomes (1-, 3-, and 5-y mortality) and corresponding Harrell’s *C*-statistics are listed in **Table S9** (**SDC**, https://links.lww.com/TXD/A861). Analogous Kaplan-Meier curves for the BAR, SOFT, and MELD scores are listed in **Figure S1** (**SDC**, https://links.lww.com/TXD/A862), **Figure S2** (**SDC**, https://links.lww.com/TXD/A863), and **Figure S3** (**SDC**, https://links.lww.com/TXD/A864).

**TABLE 3.** Comparing SOFT Cluster, BAR, SOFT, and MELD score *C*-statistics

Score	90-d mortality	1-y mortality	3-y mortality	5-y mortality	Length of stay >30 d	1-y graft failure
Cluster score—training set	0.6733 (0.659-0.688)	0.6530 (0.642-0.664)	0.6502 (0.642-0.659)	0.6426 (0.635-0.650)	0.7546 (0.746-0.763)	0.6343 (0.624-0.644)
Cluster score—validation set	0.6598 (0.639-0.680)	0.6360 (0.621-0.651)	0.6278 (0.616-0.640)	0.6299 (0.619-0.641)	0.7557 (0.744-0.768)	0.6031 (0.589-0.617)
BAR score	0.6059 (0.593-0.618)	0.5830 (0.573-0.592)	0.5421 (0.534-0.550)	0.5269 (0.520-0.534)	0.7020 (0.695-0.709)	0.5574 (0.549-0.566)
SOFT score	0.6510 (0.639-0.0.663)	0.6262 (0.617-0.635)	0.5854 (0.578-0.593)	0.5674 (0.561-0.574)	0.7203 (0.713-0.728)	0.6020 (0.594-0.610)
MELD score	0.5787 (0.566-0.591)	0.5545 (0.545-0.564)	0.5156 (0.508-0.523)	0.5010 (0.494-0.508)	0.6886 (0.681-0.696)	0.5348 (0.529-0.543)

BAR, balance of risk; MELD, Model for End-Stage Liver Disease; SOFT, Survival Outcomes Following Liver Transplantation.

## RESULTS

### Study Population

The study population included 61 231 patients. Of those 61 231 patients, 2287 patients died within 90 d of transplant, 4026 within 1 y, 6589 within 3 y, and 8227 within 5 y. Of those 61 231 patients, 6900 patients were hospitalized for >30 d posttransplant, and 2340 grafts failed within 1 y. Mean patient survival was 3.10 y, mean graft survival was 3.09 y, and mean LOS was 15.53 d. Most of these patients are male (60.6%; 39.4% female), >50 (73.6%), White (67.8%; 19.0% Hispanic, 6.5% Black), and have a body mass index >25 (74.8%). Alcohol-associated liver disease is the most common primary diagnosis for transplant candidates (39.0%), followed by metabolic dysfunction-associated steatohepatitis (20.4%).^9^ Demographic information on the training and validation sets is listed in **Table S10** (**SDC**, https://links.lww.com/TXD/A861).

### Data Entry Rate

Data entry completion for all factors is listed in **Table S1** (**SDC**, https://links.lww.com/TXD/A861). Most are well populated with a level of missingness <5%. Exceptions include recipient hepatocellular carcinoma (88.4%) and controlled donation after circulatory death (9.2%). Recipients with missing data for some risk factors were added to the reference group instead of being dropped, assuming a random distribution of missing entries to maintain the sample size.

### Univariable and Multivariable Analysis

Forty-nine significant recipient and 17 significant donor risk factors were isolated through univariable and multivariable analysis of the training set (Table 2). All the risk factors considered in the univariable and multivariable analyses are listed in **Table S1** (**SDC**, https://links.lww.com/TXD/A861). The results of multivariable analyses are listed in **Tables S2–S7** (**SDC**, https://links.lww.com/TXD/A861).

### Risk Scores

#### Ninety-day Mortality

The most significant risk factors for 90-d posttransplant mortality were recipient serum sodium of 155–160 mEq/L (OR, 4.850; CI, 2.26-10.38; *P*< 0.001), recipient serum sodium of 150–155 mEq/L (OR, 2.38; CI, 1.56-3.62; *P* < 0.001), and recipient history of 2 previous transplants (OR, 4.20; CI, 2.28-7.74; *P* < 0.001; **Table S2** [**SDC**, https://links.lww.com/TXD/A861]). The most significant protective factors included a recipient functional status of independence with minor symptoms based on Karnofsky scores (OR, 0.410; CI, 0.25-0.67; *P* < 0.001), donor hepatitis C diagnosis (OR, 0.80; CI, 0.64-1.00; *P* = 0.045), and cold ischemia time <6 h (OR, 0.830; CI, 0.75-0.93; *P* = 0.001; **Table S2** [**SDC**, https://links.lww.com/TXD/A861]). The area under the ROC curve (*C*-statistic) for 90-d posttransplant mortality was 0.6733 (CI, 0.65902-0.68750) for the training set and 0.6598 (CI, 0.63940-0.68013) for the validation set (Table 2).

#### One-year Mortality

The most significant risk factors for 1-y posttransplant mortality were recipient serum sodium 155–160 mEq/L (OR, 4.08; CI, 2.00-8.33; *P* < 0.001), recipient serum sodium 150–155 mEq/L (OR, 1.80; CI, 1.23-2.63; *P* < 0.001), and donor blood pH <7.0 (OR, 4.92; CI, 1.48-16.39; *P* = 0.01; **Table S3** [**SDC**, https://links.lww.com/TXD/A861]). The most significant protective factors included a recipient functional status of independence with mild symptoms (OR, 0.57; CI, 0.42-0.77; *P* < 0.001), recipient functional status of independence with moderate symptoms (OR, 0.72; CI, 0.61-0.86; *P* < 0.001), and recipient functional status of ability to care for oneself but inability to continue normal activity based on Karnofsky scores (OR, 0.80; CI, 0.70-0.92; *P* = 0.001; **Table S3** [**SDC**, https://links.lww.com/TXD/A861]). The area under the ROC curve for 1-y posttransplant mortality was 0.6530 (CI, 0.64189-0.66406) in the training set and 0.6360 (CI, 0.62066-0.65139) in the validation set (Table 2).

#### Three-year Mortality

The most significant risk factors for 3-y posttransplant mortality were recipient history of 2 previous transplants (OR, 3.72; CI, 2.29-6.04; *P* < 0.001), history of 1 previous transplant (OR, 2.05; CI, 1.18-3.57; *P* = 0.01), and recipient serum sodium 155–160 mEq/L (OR, 3.66; CI, 1.86-7.22; *P* < 0.001; **Table S4** [**SDC**, https://links.lww.com/TXD/A861]). The most significant protective factors included recipient functional status of independence with mild symptoms (OR, 0.64; CI, 0.52-0.79; *P* < 0.001), recipient functional status of independence with moderate symptoms (OR, 0.76; CI, 0.67-0.89; *P* < 0.001), and national allocation (OR, 0.85; CI, 0.77-0.93; *P* = 0.001; **Table S4** [**SDC**, https://links.lww.com/TXD/A861]). The area under the ROC curve for 3-y posttransplant mortality was 0.6502 (CI, 0.64161-0.65883) in the training set and 0.6278 (CI, 0.61552-0.64001) in the validation set (Table 2).

#### Five-year Mortality

The most significant risk factors for 5-y posttransplant mortality were recipient history of 2 previous transplants (OR, 3.43; CI, 2.15-5.48; *P* < 0.001), recipient serum sodium 155–160 mEq/L (OR, 3.01; CI, 1.53-5.94; *P* < 0.001), and recipient age >65 (OR, 1.60; CI, 1.48-1.73; *P* < 0.001; **Table S5** [**SDC**, https://links.lww.com/TXD/A861]). The most significant protective factors included recipient functional status of independence with mild symptoms (OR, 0.68; CI, 0.56-0.81; *P* < 0.001), recipient functional status of independence with moderate symptoms (OR, 0.79; CI, 0.71-0.89; *P* < 0.001), and national allocation (OR, 0.68; CI, 0.62-0.74; *P* < 0.001; **Table S5** [**SDC**, https://links.lww.com/TXD/A861]). The area under the ROC curve for 5-y posttransplant mortality was 0.6426 (CI, 0.63485-0.65034) for the training set and 0.6299 (CI, 0.61893-0.64087) for the validation set (Table 2).

#### LOS >30 d

The most significant risk factors for LOS >30 d were recipient history of 2 previous transplants (OR, 2.70; CI, 1.48-4.92; *P* = 0.001), recipient who’s ventilator-dependent pretransplant (OR, 2.11; CI, 1.77-2.52; *P* < 0.001), and recipient admitted to the ICU pretransplant (OR, 1.95; CI, 1.68-2.27; *P* < 0.001; **Table S6** [**SDC**, https://links.lww.com/TXD/A861]). The most significant protective factors included recipient functional status of independence with moderate symptoms (OR, 0.66; CI, 0.50-0.87; *P* = 0.003), recipient functional status of ability to care for oneself but inability to continue normal activity (OR, 0.57; CI, 0.50-0.68; *P* < 0.001), and recipient working for income (OR, 0.65; CI, 0.58-0.72; *P* < 0.001; **Table S6** [**SDC**, https://links.lww.com/TXD/A861]). The area under the ROC curve for LOS >30 d was 0.7546 (CI, 0.74629-0.76299) for the training set and 0.7557 (CI, 0.74387-0.76761) for the validation set (Table 2).

#### One-year Graft Failure

The most significant risk factors for 1-y graft failure were serum sodium 155–160 mEq/L (OR, 2.85; CI, 1.40-5.79; *P* = 0.004), serum sodium 150–155 mEq/L (OR, 1.51; CI, 1.05-2.18; *P* = 0.028), and controlled donation (OR, 1.68; CI, 1.49-1.89; *P* < 0.001; **Table S7** [**SDC**, https://links.lww.com/TXD/A861]). The most significant protective factors included recipient functional status of independence with moderate symptoms (OR, 0.79; CI, 0.69-0.91; *P* = 0.001), recipient functional status of independence with mild symptoms (OR, 0.73; CI, 0.58-0.93; *P* = 0.01), and cold ischemia time <6 h (OR, 0.79; CI, 0.74-0.85; *P* < 0.001; **Table S7** [**SDC**, https://links.lww.com/TXD/A861]). The area under the ROC curve for 1-y graft failure was 0.6343 (CI, 0.62432-0.64434) for the training set and 0.6031 (CI, 0.58904-0.61723) for the validation set (Table 2).

### SOFT Cluster Score

Table 2 details the SOFT Cluster score, with each of the risk and protective factors listed with the corresponding number of points allotted to the overall risk score for each of the 6 outcomes. Table 4 demonstrates the Cluster score for 6 sample patients. Patients 1 and 2 demonstrate patients with homogenous Cluster scores where risk is decreased or increased, respectively, across all 6 measures of patient outcomes. Patients 3 and 4 represent patients with increased risk in more acute outcomes (90-d and 1-y mortality and LOS) with decreased risk of chronic outcomes (3- and 5-y mortality and graft failure), while patients 5 and 6 represent the inverse scenario. These sample patients highlight how the Cluster score creates a more comprehensive assessment of patient risk surrounding transplant outcomes.

**TABLE 4.** SOFT Cluster scores for 6 sample patients

Sample patient	90-d mortality score	1-y mortality score	3-y mortality score	5-y mortality score	Prolonged LOS	1-y graft failure score	Outcome
1	0.46	0.56	0.76	0.91	0.77	0.59	A
2	2.01	1.76	1.84	1.67	3.16	1.76	D
3	1.40	1.12	0.77	0.67	3.48	0.84	A
4	1.75	1.27	0.93	1.01	3.14	1.22	L
5	1.04	0.85	1.21	1.36	0.54	0.99	A
6	0.64	0.80	1.29	1.27	0.55	1.09	A

A, alive; D, dead; L, lost to follow-up; SOFT, Survival Outcomes Following Liver Transplantation.

### Comparison With BAR, SOFT, and MELD Scores

Discrimination of the SOFT Cluster score was compared with BAR, SOFT, and MELD scores across 6 clinical endpoints using DeLong test (**Table S8**, **SDC**, https://links.lww.com/TXD/A861). For 90-d mortality, the SOFT Cluster score demonstrated the highest discrimination (AUC = 0.669; 95% CI, 0.657-0.680), exceeding BAR (AUC = 0.606; *P* < 0.001), SOFT (AUC = 0.651; *P* = 0.002), and MELD (AUC = 0.579; *P* < 0.001). Similar findings were observed for 1-y mortality (SOFT Cluster AUC = 0.647), 3-y mortality (AUC = 0.643), and 5-y mortality (AUC = 0.638), with the SOFT Cluster score demonstrating significantly greater discrimination than all comparator scores for each endpoint (all DeLong *P* < 0.001). When compared with the BAR score, which was previously used by Zakareya et al^10^ to predict survival at 1, 3, and 5 y after transplant, the SOFT Cluster score had a higher *C*-statistic for these measures.

For LOS >30 d, the SOFT Cluster score again showed the strongest discrimination (AUC = 0.755; 95% CI, 0.748–0.762), outperforming BAR (AUC = 0.702), SOFT (AUC = 0.720), and MELD (AUC = 0.689; all *P* < 0.001). Similarly, for 1-y graft failure, the SOFT Cluster score achieved higher discrimination (AUC = 0.624; 95% CI, 0.616–0.632) compared with BAR (AUC = 0.557), SOFT (AUC = 0.602), and MELD (AUC = 0.535; all *P* < 0.001). Across all 6 endpoints, the SOFT Cluster score demonstrated higher observed discrimination than the BAR, SOFT, and MELD scores. This trend was also observed with the use of Cox proportional hazards ratios and Harrell’s *C*-statistics (**Table S9**, **SDC**, https://links.lww.com/TXD/A861).

## DISCUSSION

**FIGURE 1.**
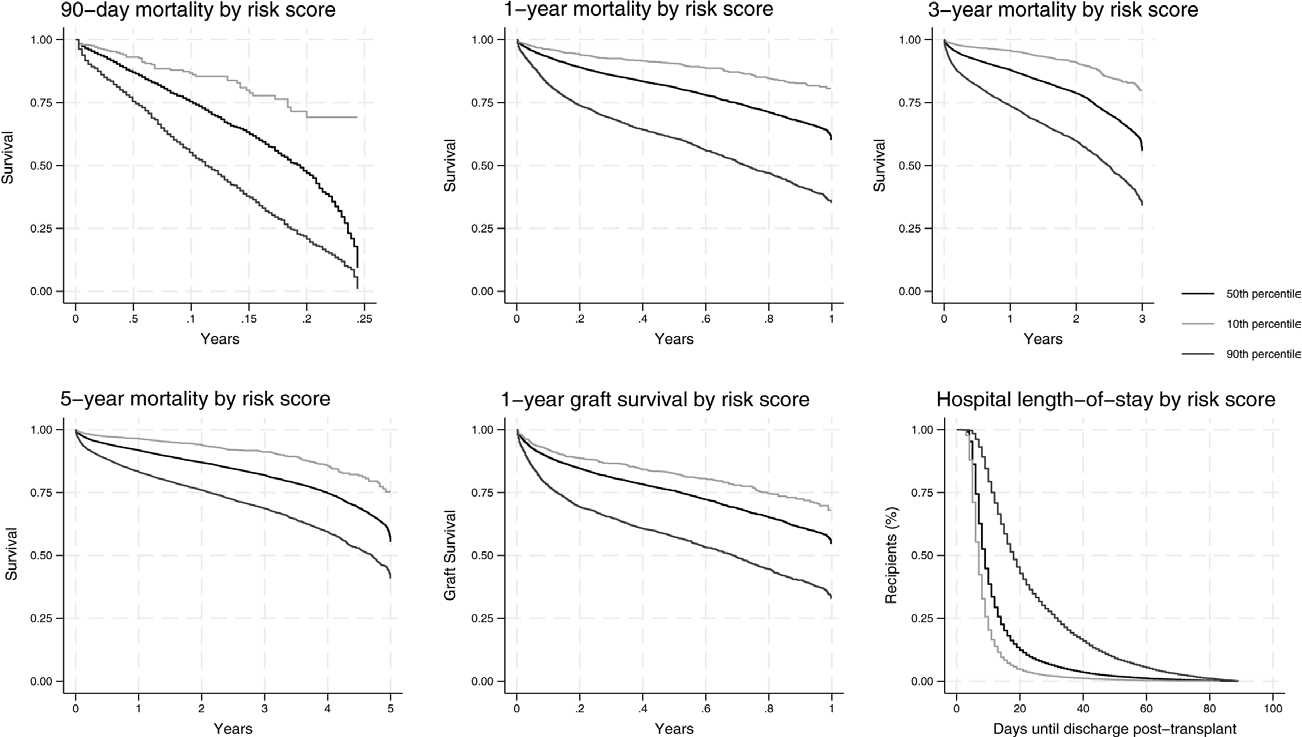
Kaplan-Meier curves for posttransplant outcomes by risk score group. The y-axis is the percentage survival of the total sample of recipients, and the x-axis is the months elapsed posttransplant. Patients are divided into the 10th, 50th, and 90th percentiles for each of the 6 risk scores that comprise their Survival Outcomes Following Liver Transplantation (SOFT) Cluster score.

In this retrospective cohort study, the OPTN liver transplant database was used to analyze donor and recipient factors that contribute to posttransplant mortality, prolonged LOS, and graft failure. Risk index equations were generated for 6 different outcomes: patient mortality at 90-d, 1-y, 3-y, and 5-y posttransplant, as well as hospital LOS >30 d, and graft failure at 1-y posttransplant. Kaplan-Meier survival curves for each of these scoring systems are listed in Figure 1 with patients split into the 10th, 50th, and 90th percentiles.

Factors associated with a homogenous increase in risk include recipient hypernatremia, history of previous transplants, previous abdominal surgery, low pretransplant functional status, ventilator dependence pretransplant, portal vein thrombosis pretransplant, and recipient age >65. Recipient hypernatremia increased risk scores across all 6 domains of the SOFT Cluster score. McDonald et al^11^ also found that recipient hypernatremia increased mortality risk in both the acute and chronic phase of recovery, as well as a higher risk of having prolonged LOS and graft failure. They reason that hypernatremia likely serves as an indicator of poor prognosis rather than a causative agent itself,^11^ while Leise et al^12^ argued that this conclusion can be expanded to use hypernatremia as evidence of the quality of a patient’s intensive care. While this study found that hypernatremic patients were sicker in general, with increased need for dialysis, ventilators, and higher MELD scores, the effect of hypernatremia on increased mortality risk remained significant when controlling for these potential confounding variables.^12^ Clarifying the role of increased serum sodium on posttransplant outcomes warrants further investigation given the strength of this demonstrated effect across all metrics in this analysis.

Protective factors associated with homogenous decrease in risk include high pretransplant functional status, working for income, and younger recipient age at transplant. Historically, younger recipient age has been associated with improved outcomes. Gómez-Gavara et al^13^ found a similar increase in posttransplant mortality but not graft failure in older patients with hepatocellular carcinoma. This difference in risk might be attributed to older patients having lower rates of retransplantation, which was also identified as a significant risk factor in the present analysis. Because age impacts such a broad variety of clinical conditions, it is more appropriately used within the context of a patient’s risk profile rather than as an isolated metric. This notion is supported by Niazi et al^14^ who demonstrated heterogeneity of risk across both younger and older transplant patients and argued for a comprehensive examination of risk rather than using age as a cutoff given its relationship to other clinical conditions. Ultimately, the factors that affect risk across all 6 domains of the SOFT Cluster score are still most useful to clinicians when considered within in the context of a given patient’s clinical condition.

**FIGURE 2.**
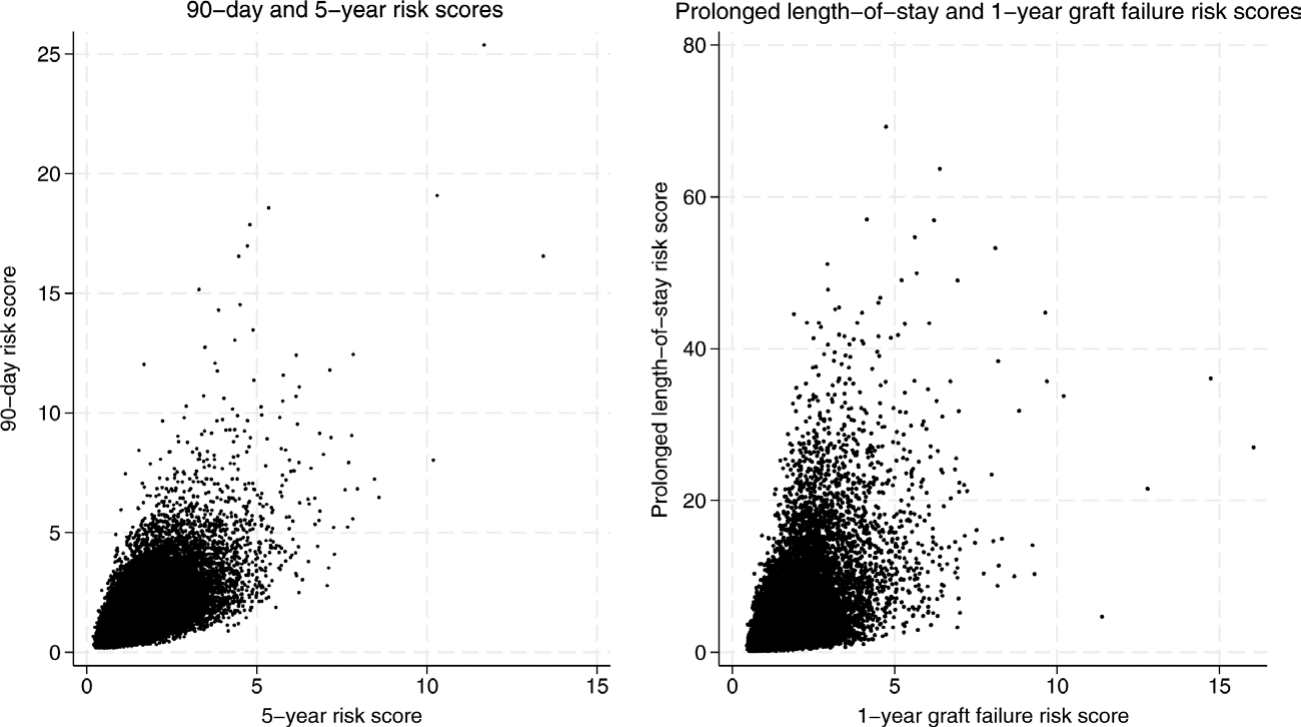
Scatter plots of short-term and long-term outcomes by risk score. The y-axis represents a short-term measure of posttransplant outcomes, and the x-axis represents a long-term metric. The scatter plots depict the spread and variability between these scores for the total sample of recipients.

Conversely, some factors included in the SOFT Cluster score had differential effects on short- and long-term metrics. Figure 2 displays 2 scatter plots that depict the spread and variability between short-term and long-term risk scores for the total sample of recipients. Acute factors like cold ischemia time were more influential in predicting short-term outcomes. Lozanovski et al^15^ similarly found that prolonged cold ischemia time only increased patient mortality during the first year after transplantation, likely because the graft has recovered from any effects of ischemia-reperfusion injury by this time. Other measures like national graft allocation or increased distance from the donor hospital had a greater impact on long-term risk. Imaoka et al^16^ found an increase in risk of negative postoperative outcomes associated with grafts traveling longer distances; however, they observed this risk only when assessing 90-d graft survival. While Imaoka et al^16^ cite reperfusion injury as 1 possible cause for their findings, their discussion of how marginal grafts may travel increased distances to more aggressive transplant centers following rejection by regional centers may represent another explanation for an increased risk of negative outcomes in the long term. Region was incorporated as a predictive factor because intention-to-treat analysis has previously elucidated significant differences in mortality rates by region because of geographic disparities in liver transplantation.^17^

Additionally, a recipient MELD >40 increased their risk of prolonged LOS and decreased risk of 1-y graft failure, but it was not a significant predictor of posttransplant mortality. Bleszynski et al^18^ reported similar findings where a MELD score >35 was not a significant predictor of mortality, only for prolonged LOS and graft failure at 1 and 3 y posttransplant. Others argue that better graft selection, decreased cold ischemia time, and ABO compatibility may contribute to improved outcomes in high MELD patients.^19^

There is still no consensus on how to accurately assess patient risk and which donor and recipient factors should be considered.^6^ Baganate et al^20^ highlights the need for a greater understanding of postoperative mortality rather than relying on 1-y mortality as a single reflection of efficacy to better understand cause- and timing-specific causes of death. The SOFT Cluster score is a proof-of-concept model that addresses this need by taking a multifaceted approach to characterizing patient risk and providing more insight into a patient’s risk of adverse postoperative outcomes. Because there is variation in which factors are significant predictors of short-term and long-term metrics, the SOFT Cluster score provides a more robust understanding of patient risk by generating a profile of scores as opposed to a single measure. While the SOFT Cluster *C*-statistics are not impressive for current use in clinical practice, this model represents a platform upon which more accurate scores can be built in the future. This methodology represents an advantage over existing models because it considers how factors that predict short-term outcomes are different from those that predict long-term outcomes. It is a proof-of-concept model with significant potential for future expansion and generation of more accurate risk indices in the future. The complexity of the SOFT Cluster score represents a limitation of its utility, as it compromises ease of use for greater coverage of donor and recipient characteristics. However, the magnitude of this limitation is lessened by the advent of large-language models and the artificial intelligence, and incorporation of clinical risk scores into the electronic medical record, which can better facilitate the clinical workflow of future iterations of the SOFT Cluster model.

This analysis is limited by variability in the degree of data entry between different hospitals. Analysis of center-level characteristics like transplant volume and institutional guidelines was not possible because of the nature of this database, which represents a significant limitation of this model for predicting endpoints like LOS. Another limitation of the SOFT Cluster score is fully characterizing 3- and 5-y survival, as pretransplant variables cannot fully account for a long-term complex clinical picture. The use of logistic regression for long-term outcomes limits discrimination between factors associated with early survival and those exerting a sustained effect over time. Although time-to-event analysis was conducted as sensitivity assessments, because logistic regression was used as the primary framework for analysis of mortality, findings involving perioperative factors in long-term mortality models should still be interpreted with caution. Other limitations include the retrospective nature of this analysis and reduced generalizability outside of the United States. One avenue for improvement of the SOFT Cluster score includes time-to-event assessment of other related etiologies of mortality in addition to graft failure. Recipient factors included in the SOFT Cluster score may also be impacted by ongoing changes to the current graft allocation system. Additionally, the increasingly widespread use of machine perfusion should be incorporated into risk scoring systems, as perfusion will likely change the future predictive ability of models built upon the SOFT Cluster score.

## CONCLUSIONS

The SOFT Cluster score represents a novel approach to assessment of posttransplant risk through analyzing a patient’s risk of postoperative mortality at 4 different timepoints, risk of prolonged LOS, and risk of graft failure by creating a comprehensive profile generated through consideration of varied donor and recipient characteristics. Unlike previously established transplant risk scores, the SOFT Cluster score offers a more holistic perspective on evaluating liver transplant patients by approaching risk assessment from multiple angles rather than a single score. This proof-of-concept model holds the potential to serve as a valuable conceptual framework for multifactorial interpretation of time-stratified postoperative risk.

## Supplementary Material


