# Supplementary material for: The SOFT Cluster Score as a Multifaceted Predictive Model for Postoperative Outcomes
Source: Transplant Direct. 2026 May 6;12(6):e1951. doi: 10.1097/TXD.0000000000001951 (PMC13155515; doi:10.1097/TXD.0000000000001951)
Supplement: Supplementary file 1 [file txd-12-e1951-s001.pdf]

Table S1: Variables considered for univariable and multivariable analysis

Recipient Risk Factors	Entry completion	Percent of patients	Donor Risk Factors	Percent entry filled	Percent of patients
ABO incompatible transplant	100.0%	1.3%	Age < 10	100.0%	0.5%
ABO compatible transplant	100.0%	6.5%	Age 10-15	100.0%	1.4%
Age 18-30	100.0%	4.0%	Age 15-20	100.0%	7.4%
Age 60-65	100.0%	20.5%	Age 20-30	100.0%	19.1%
Age > 65	100.0%	21.6%	Age 45-55	100.0%	19.4%
Albumin 2.0-2.5 g/dL	100.0%	13.8%	Age 55-60	100.0%	9.6%
Albumin 1.5-2.0 g/dL	100.0%	4.1%	Age 60-70	100.0%	11.9%
Albumin <1.5 g/dL	100.0%	0.6%	Age >70	100.0%	3.8%
Ascites pretransplant	100.0%	75.0%	Cold ischemia time 0-6 hours	99.4%	55.7%
Bacterial peritonitis pretransplant	99.9%	9.6%	Cold ischemia time 12-14 hours	99.4%	1.1%
Body mass index 30-35	99.8%	24.0%	Cold ischemia time >14 hours	99.4%	1.1%
Body mass index 35-40	99.8%	11.5%	Creatinine 1.5-2.0 mg/dL	99.7%	10.0%
Body mass index >40	99.8%	5.4%	Creatinine >2.0 mg/dL	99.7%	24.5%
Education level – Dropped out	99.9%	5.3%	Controlled donation after circulatory death	9.2%	9.2%
Education level – High school diploma or GED	99.9%	38.7%	Distance from donor hospital 500-1000 mi	100.0%	4.4%
Education level – Technical school	99.9%	25.3%	Distance from donor hospital >1000 mi	100.0%	1.3%
Education level – bachelor’s degree	99.9%	20.5%	Race – African American	100.0%	18.2%
Education level – graduate degree	99.9%	7.7%	Hepatitis C	99.9%	8.4%
Encephalopathy at transplant	100.0%	13.4%	Height difference at	99.8%	1.8%

			transplant 30-60 cm		
Race – African American	100.0%	7.6%	Height difference at transplant > 60 cm	99.8%	0.1%
Hepatitis C	100.0%	10.6%	Weight difference at transplant 45-70 kg	99.9%	5.0%
Hepatocellular carcinoma	88.4%	16.3%	Weight difference at transplant > 70 kg	99.9%	1.0%
MELD score 30-35	100.0%	13.0%	Weight difference at transplant -45 to -70 kg	99.9%	2.6%
MELD score 35-40	100.0%	10.0%	Weight difference at transplant > -70 kg	99.9%	0.6%
MELD score >40	100.0%	9.2%	Blood pH 7.1-7.2	99.6%	0.8%
Serum sodium 130-135 mEq/L	100.0%	28.0%	Blood pH 7.0-7.1	99.6%	0.2%
Serum sodium 125-130 mEq/L	100.0%	12.3%	Blood pH < 7.0	99.6%	0.1%
Serum sodium < 125 mEq/L	100.0%	4.2%	AST/SGOT < 90 U/L	99.7%	9.6%
Serum sodium 145-150 mEq/L	100.0%	2.4%	AST/SGOT > 140 U/L	99.7%	13.7%
Serum sodium 150-155 mEq/L	100.0%	0.5%	ALT/SGPT < 60 U/L	99.7%	14.0%
Serum sodium 155-160 mEq/L	100.0%	0.1%	ALT/SGPT > 100 U/L	99.7%	19.1%
Serum sodium > 160 mEq/L	100.0%	0.0%	Regional allocation	100.0%	29.9%
Functional status at transplant: 2010	99.8%	4.9%	National allocation	100.0%	16.6%
Functional status at transplant: 2020	99.8%	14.2%	Foreign allocation	100.0%	0.0%
Functional status at transplant: 2030	99.8%	11.0%	Total bilirubin 1.0-1.8 mg/dL	99.7%	18.7%
Functional status at transplant: 2040	99.8%	13.0%	Total bilirubin >1.8 mg/dL	99.7%	8.3%

Functional status at transplant: 2050	99.8%	13.3%	
Functional status at transplant: 2060	99.8%	12.7%	
Functional status at transplant: 2070	99.8%	14.5%	
Functional status at transplant: 2080	99.8%	10.7%	
Functional status at transplant: 2090	99.8%	3.6%	
Functional status at transplant: 2100	99.8%	0.9%	
INR 2.0-2.5	100.0%	16.5%	
INR 2.5-3.0	100.0%	9.8%	
INR 3.0-3.5	100.0%	5.4%	
INR 3.5-4.0	100.0%	2.2%	
INR > 4.0	100.0%	3.8%	
Life support pre-transplant	99.9%	8.6%	
ICU pre-transplant	99.9%	15.2%	
Hospital admission pre-transplant	99.9%	21.5%	
Dialysis prior to transplant	99.6%	12.8%	
One previous transplant	100.0%	3.7%	
Two previous transplants	100.0%	0.3%	
Three previous transplants	100.0%	0.1%	
Early re-transplant (less than 90 days)	100.0%	1.4%	
Two early re-transplants	100.0%	0.1%	
Late re-transplant (over 90 days)	100.0%	2.1%	
Two late re-transplants	100.0%	3.5%	
Ventilator-dependent pre-transplant	100.0%	4.4%	
Portal vein thrombosis at transplant	99.8%	14.0%	

Portal vein thrombosis at registration	99.9%	8.2%	
Primary payment – Medicaid	99.8%	16.4%	
Primary payment – private insurance	99.8%	51.5%	
Previous abdominal surgery	99.8%	48.0%	
OPTN region of transplant registration – Region 1	100.0%	4.0%	
Region 2	100.0%	10.7%	
Region 3	100.0%	16.9%	
Region 4	100.0%	10.2%	
Region 5	100.0%	15.5%	
Region 6	100.0%	3.1%	
Region 7	100.0%	8.1%	
Region 8	100.0%	6.2%	
Region 9	100.0%	5.7%	
Region 10	100.0%	9.5%	
Region 11	100.0%	10.3%	
Total bilirubin <2.0 mg/dL	100.0%	28.7%	
Total bilirubin 8.0-16.0 mg/dL	100.0%	14.4%	
Total bilirubin 16.0-32.0 mg/dL	100.0%	13.7%	
Total bilirubin >32.0 mg/dL	100.0%	6.7%	
TIPS at transplant	99.8%	9.8%	
Working for income at transplant	99.7%	20.7%	

ABO = A, B, O blood group system; GED = general educational development; MELD = model for end-stage liver disease; AST/SGOT = aspartate aminotransferase/serum glutamic-oxaloacetic transaminase; ALT/SGPT = alanine aminotransferase/serum glutamic-pyruvic transaminase; INR = international normalized ratio; ICU = intensive care unit; OPTN = organ procurement and transplantation network; TIPS = transjugular intrahepatic portosystemic shunt.

Table S2: 90-day Multivariable Logistic Regression

**Recipient Factors**	**OR**	**95% CI**	***p*-value**
ABO compatible transplant	1.15	(0.96, 1.38)	0.136

Age > 65	1.53	(1.34,1.74)	0
Albumin 2.0-2.5 g/dL	1.17	(1.01, 1.35)	0.032
BMI 35-40	1.33	(1.14,1.55)	0
BMI >40	1.41	(1.14, 1.75)	0.002
Dialysis prior to transplant	1.4	(1.17, 1.68)	0
Education level - technical school	0.86	(0.76, 0.97)	0.016
Encephalopathy at transplant	1.15	(1.00, 1.33)	0.05
African American	1.21	(1.01, 1.45)	0.038
Hepatocellular carcinoma	0.89	(0.74, 1.07)	0.217
MELD score 30-35	0.96	(0.79, 1.17)	0.704
MELD score 35-40	1.02	(0.81, 1.30)	0.844
Serum sodium 130-135 mEq/L	0.97	(0.86, 1.10)	0.674
Serum sodium 125-130 mEq/L	1.02	(0.86, 1.22)	0.804
Serum sodium 145-150 mEq/L	1.59	(1.23, 2.05)	0
Serum sodium 150-155 mEq/L	2.38	(1.56, 3.62)	0
Serum sodium 155-160 mEq/L	4.84	(2.26, 10.38)	0
Serum sodium > 160 mEq/L	3.04	(0.65, 14.31)	0.159
Functional status at transplant: 2010	1.84	(1.44, 2.35)	0
Functional status at transplant: 2020	1.47	(1.23, 1.75)	0
Functional status at transplant: 2050	0.93	(0.77, 1.13)	0.477
Functional status at transplant: 2060	0.85	(0.70, 1.05)	0.127
Functional status at transplant: 2070	0.9	(0.74, 1.09)	0.273
Functional status at transplant: 2080	0.8	(0.64, 1.01)	0.062
Functional status at transplant: 2090	0.41	(0.25, 0.67)	0
INR > 4.0	1.12	(0.87, 1.45)	0.359
Life support pre-transplant	1.1	(0.87, 1.40)	0.41
ICU pre-transplant	1.2	(0.99, 1.45)	0.06
One previous transplant	2.65	(1.27, 5.56)	0.01
Two previous transplants	4.2	(2.28, 7.74)	0
Late re-transplant	1.01	(0.47, 2.19)	0.982
Early re-transplant	0.68	(0.31, 1.49)	0.336
Two late re-transplants	1	NA	0
Two early re-transplants	0.71	(0.21, 2.33)	0.567
Ventilator-dependent pre-transplant	1.17	(0.90, 1.53)	0.237
Portal vein thrombosis at registration	0.91	(0.74, 1.12)	0.367
Portal vein thrombosis at transplant	1.44	(1.22, 1.69)	0
Primary payment - private insurance	0.92	(0.82, 1.03)	0.14
Previous abdominal surgery	1.32	(1.18, 1.47)	0
Total bilirubin < 2.0 mg/dL	1.18	(1.02, 1.37)	0.026

Total bilirubin 16.0-32.0 mg/dL	0.9	(0.76, 1.06)	0.201
Total bilirubin > 32.0 mg/dL	0.95	(0.76, 1.19)	0.654
Working for income	0.87	(0.75, 1.02)	0.082
**Donor Factors**	**OR**	**95% CI**	***p*-value**
Cold ischemia time 0-6 hours	0.83	(0.75, 0.93)	0.001
Cold ischemia time 12-14 hours	1.72	(1.17, 2.53)	0.006
Controlled donation	1.1	(0.90, 1.34)	0.337
Donor Positive Hepatitis C Serology	0.8	(0.64, 1.00)	0.045
Blood pH < 7.0	5.68	(1.49, 21.68)	0.011
Regional allocation	0.9	(0.80, 1.01)	0.079
Weight difference at transplant 45-70 kg	1.1	(0.87, 1.40)	0.402
Weight difference at transplant -45 to -70 kg	1.54	(1.15, 2.06)	0.004

ABO = A, B, O blood group system; BMI = body mass index; MELD = model for end-stage liver disease; INR = international normalized ratio; ICU = intensive care unit.

Table S3: 1-year Multivariable Logistic Regression

Recipient Factors	OR	95% CI	*p-value*
ABO compatible transplant	1.09	(0.94, 1.27)	0.236
Age 18-30	0.66	(0.52, 0.84)	0.001
Age > 65	1.64	(1.48, 1.83)	0
Albumin 2.0-2.5 g/dL	1.16	(1.03, 1.29)	0.01
Ascites at transplant	1.05	(0.95, 1.16)	0.365
BMI 35-40	1.19	(1.05, 1.34)	0.006
BMI > 40	1.18	(0.99, 1.41)	0.063
Dialysis prior to transplant	1.33	(1.17, 1.51)	0
Education level - high school	1.09	(1.00, 1.18)	0.049
Encephalopathy at transplant	1.21	(1.09, 1.36)	0.001
African American	1.18	(1.02, 1.36)	0.023
Hepatocellular carcinoma	1.17	(1.03, 1.33)	0.015
MELD 30-35	0.89	(0.78, 1.02)	0.089
Serum sodium 130-135 mEq/L	0.94	(0.85, 1.03)	0.206
Serum sodium 125-130 mEq/L	1.01	(0.88, 1.16)	0.874
Serum sodium 145-150 mEq/L	1.45	(1.18, 1.80)	0
Serum sodium 150-155 mEq/L	1.8	(1.23, 2.63)	0.003
Serum sodium 155-160 mEq/L	4.08	(2.00, 8.33)	0
Functional status at transplant: 2010	1.47	(1.21, 1.78)	0
Functional status at transplant: 2020	1.27	(1.11, 1.45)	0
Functional status at transplant: 2060	0.78	(0.68, 0.90)	0.001
Functional status at transplant: 2070	0.8	(0.70, 0.92)	0.001

Functional status at transplant: 2080	0.72	(0.61, 0.86)	0
Functional status at transplant: 2090	0.57	(0.42, 0.77)	0
INR > 4.0	0.95	(0.78, 1.17)	0.65
Life support pre-transplant	1.14	(0.94, 1.38)	0.183
ICU pre-transplant	1.12	(0.97, 1.31)	0.131
One previous transplant	1.74	(0.86, 3.52)	0.123
Two previous transplants	4.39	(2.59, 7.43)	0
Late re-transplant	1.4	(0.68, 2.92)	0.362
Early re-transplant	1.2	(0.57, 2.51)	0.632
Two late re-transplants	1	NA	0
Two early re-transplants	0.44	(0.14, 1.42)	0.17
Ventilator-dependent pre-transplant	1.31	(1.05, 1.62)	0.015
Portal vein thrombosis at registration	0.99	(0.85, 1.16)	0.933
Portal vein thrombosis at transplant	1.34	(1.18, 1.51)	0
Primary payment - private insurance	0.92	(0.83, 1.02)	0.117
Primary payment - Medicaid	0.88	(0.77, 1.00)	0.057
Previous abdominal surgery	1.17	(1.08, 1.27)	0
OPTN transplant region 1	1.23	(1.02, 1.49)	0.03
OPTN transplant region 2	1.28	(1.13, 1.45)	0
OPTN transplant region 6	0.83	(0.63, 1.09)	0.176
Total bilirubin 16.0-32.0 mg/dL	0.92	(0.82, 1.04)	0.203
Total bilirubin > 32.0 mg/dL	1.06	(0.91, 1.24)	0.43
TIPS at transplant	1.17	(1.03, 1.33)	0.016
Working for income	0.8	(0.71, 0.90)	0
**Donor Factors**	**OR**	**95% CI**	***p-value***
Cold ischemia time 0-6 hours	0.83	(0.77, 0.90)	0
Blood pH < 7.0	4.92	(1.48, 16.39)	0.009
ALT/SGPT > 100 U/L	0.92	(0.83, 1.02)	0.111
Regional allocation	0.95	(0.86, 1.03)	0.218
Weight difference at transplant 45-70kg	1.07	(0.89, 1.29)	0.463

ABO = A, B, O blood group system; BMI = body mass index; MELD = model for end-stage liver disease; INR = international normalized ratio; ICU = intensive care unit; OPTN = organ procurement and transplantation network; TIPS = transjugular intrahepatic portosystemic shunt; ALT/SGPT = alanine aminotransferase/serum glutamic-pyruvic transaminase.

Table S4: 3-year multivariable logistic regression

Recipient Factors	OR	95% CI	*p*-value
ABO compatible transplant	1.04	(0.92, 1.18)	0.516
Age 18-30	0.77	(0.64, 0.93)	0.006

Age 60-65	1.43	(1.32, 1.56)	0
Age >65	1.69	(1.55, 1.84)	0
Albumin 2.0-2.5 g/dL	1.17	(1.06, 1.28)	0.001
BMI 35-40	1.16	(1.05, 1.27)	0.003
Positive Hepatitis C Serology	1.06	(0.95, 1.17)	0.296
Dialysis prior to transplant	1.3	(1.15, 1.47)	0
Education level - high school	1.13	(1.04, 1.21)	0.002
Education level - bachelor's degree	0.98	(0.90, 1.08)	0.738
Education level - graduate degree	0.96	(0.84, 1.10)	0.563
Encephalopathy at transplant	1.17	(1.06, 1.28)	0.001
African American	1.39	(1.25, 1.56)	0
Hepatocellular carcinoma	1.26	(1.14, 1.39)	0
MELD 35-40	0.92	(0.82, 1.05)	0.215
MELD >40	0.97	(0.84, 1.12)	0.643
Serum sodium 130-135 mEq/L	1.03	(0.95, 1.11)	0.488
Serum sodium 125-130 mEq/L	0.98	(0.88, 1.10)	0.774
Serum sodium 120-125 mEq/L	0.94	(0.78, 1.13)	0.518
Serum sodium 145-150 mEq/L	1.41	(1.18, 1.69)	0
Serum sodium 150-155 mEq/L	1.72	(1.23, 2.41)	0.002
Serum sodium 155-160 mEq/L	3.66	(1.86, 7.22)	0
Functional status at transplant: 2010	1.47	(1.24, 1.74)	0
Functional status at transplant: 2020	1.26	(1.13, 1.41)	0
Functional status at transplant: 2060	0.8	(0.72, 0.89)	0
Functional status at transplant: 2070	0.85	(0.76, 0.94)	0.001
Functional status at transplant: 2080	0.76	(0.67, 0.89)	0
Functional status at transplant: 2090	0.64	(0.52, 0.79)	0
INR 2.0-2.5	0.89	(0.81, 0.98)	0.014
Life support pre-transplant	1.1	(0.93, 1.31)	0.243
ICU pre-transplant	1.05	(0.92, 1.19)	0.455
One previous transplant	2.05	(1.18, 3.57)	0.011
Two previous transplants	3.72	(2.29, 6.04)	0
Late re-transplant	1.01	(0.57, 1.81)	0.961
Early re-transplant	1.01	(0.56, 1.82)	0.984
Two late re-transplants	1	NA	0
Two early re-transplants	0.77	(0.28, 2.11)	0.616
Ventilator-dependent pre-transplant	1.28	(1.06, 1.55)	0.012
Portal vein thrombosis at registration	0.98	(0.86, 1.12)	0.753
Portal vein thrombosis at transplant	1.29	(1.16, 1.42)	0
Primary payment - private insurance	0.89	(0.83, 0.96)	0.002

Previous abdominal surgery	1.13	(1.05, 1.20)	0
OPTN transplant region 2	1.26	(1.15, 1.39)	0
OPTN transplant region 6	0.85	(0.69, 1.05)	0.124
Total bilirubin < 2.0 mg/dL	1.21	(1.11, 1.32)	0
Total bilirubin 16.0-32.0 mg/dL	0.95	(0.86, 1.05)	0.349
Total bilirubin > 32.0 mg/dL	1.14	(0.99, 1.31)	0.06
TIPS at transplant	1.19	(1.07, 1.32)	0.001
Working for income	0.8	(0.73, 0.88)	0
**Donor Factors**	**OR**	**95% CI**	***p*-value**
Donor age 65-70	1.17	(1.06, 1.29)	0.001
Donor age >70	1.23	(1.05, 1.44)	0.01
Cold ischemia time 0-6 hours	0.87	(0.81, 0.92)	0
Cold ischemia time >14 hours	0.67	(0.46, 0.97)	0.036
Regional allocation	0.93	(0.86, 1.00)	0.046
National allocation	0.85	(0.77, 0.93)	0.001

ABO = A, B, O blood group system; BMI = body mass index; MELD = model for end-stage liver disease; INR = international normalized ratio; ICU = intensive care unit; OPTN = organ procurement and transplantation network; TIPS = transjugular intrahepatic portosystemic shunt.

Table S5: 5-year multivariable logistic regression

Recipient Factors	OR	95% CI	*p*-value
ABO compatible transplant	1.03	(0.92, 1.16)	0.57
Age 18-30	0.76	(0.64, 0.90)	0
Age 60-65	1.44	(1.34, 1.55)	0
Age > 65	1.6	(1.48, 1.73)	0
Albumin 2.0-2.5 g/dL	1.15	(1.06, 1.26)	0
BMI 35-40	1.11	(1.01, 1.21)	0.03
Positive Hepatitis C Serology	1.13	(1.04, 1.24)	0.01
Dialysis prior to transplant	1.22	(1.09, 1.36)	0
Education level - high school	1.1	(1.03, 1.18)	0
Education level - bachelor's degree	0.95	(0.87, 1.03)	0.21
Education level - graduate degree	0.93	(0.83, 1.05)	0.26
Encephalopathy at transplant	1.13	(1.04, 1.24)	0.01
African American	1.31	(1.18, 1.45)	0
Hepatocellular carcinoma	1.26	(1.16, 1.38)	0
MELD 35-40	1.01	(0.91, 1.11)	0.91
MELD >40	1.08	(0.95, 1.22)	0.24
Serum sodium 130-135 mEq/L	1.01	(0.94, 1.08)	0.83
Serum sodium 125-130 mEq/L	0.94	(0.85, 1.04)	0.22

Serum sodium 120-125 mEq/L	0.91	(0.77, 1.08)	0.29
Serum sodium 145-150 mEq/L	1.35	(1.14, 1.60)	0
Serum sodium 150-155 mEq/L	1.55	(1.12, 2.15)	0.01
Serum sodium 155-160 mEq/L	3.01	(1.53, 5.94)	0
Functional status at transplant: 2010	1.41	(1.20, 1.65)	0
Functional status at transplant: 2020	1.25	(1.12, 1.39)	0
Functional status at transplant: 2060	0.83	(0.75, 0.92)	0
Functional status at transplant: 2070	0.84	(0.77, 0.93)	0
Functional status at transplant: 2080	0.79	(0.71, 0.89)	0
Functional status at transplant: 2090	0.68	(0.56, 0.81)	0
INR 2.0-2.5	0.91	(0.84, 0.99)	0.04
Life support pre-transplant	1.08	(0.92, 1.27)	0.33
ICU pre-transplant	1.01	(0.88, 1.15)	0.93
Hospital admission pre-transplant	1.01	(0.92, 1.11)	0.84
One previous transplant	1.58	(0.91, 2.75)	0.11
Two previous transplants	3.43	(2.15, 5.48)	0
Late re-transplant	1.18	(0.66, 2.11)	0.57
Early re-transplant	1.33	(0.74, 2.40)	0.34
Two late re-transplants	1	NA	0
Two early re-transplants	0.67	(0.25, 1.84)	0.44
Ventilator-dependent pre-transplant	1.32	(1.10, 1.58)	0
Portal vein thrombosis at registration	0.92	(0.82, 1.04)	0.17
Portal vein thrombosis at transplant	1.31	(1.19, 1.44)	0
Primary payment - private insurance	0.88	(0.82, 0.94)	0
Previous abdominal surgery	1.14	(1.07, 1.21)	0
OPTN transplant region 1	1.14	(0.99, 1.32)	0.07
OPTN transplant region 2	1.23	(1.12, 1.35)	0
OPTN transplant region 5	0.84	(0.76, 0.91)	0
OPTN transplant region 6	0.8	(0.66, 0.96)	0.02
Total bilirubin < 2.0 mg/dL	1.26	(1.16, 1.36)	0
Total bilirubin 16.0-32.0 mg/dL	0.97	(0.88, 1.06)	0.51
Total bilirubin > 32.0 mg/dL	1.1	(0.97, 1.25)	0.14
TIPS at transplant	1.16	(1.06, 1.27)	0
Working for income	0.8	(0.73, 0.87)	0
**Donor Factors**	**OR**	**95% CI**	***p*-value**
Donor age 65-70	1.13	(1.04, 1.24)	0.01
Donor age >70	1.25	(1.08, 1.44)	0
Cold ischemia time 0-6 hours	0.83	(0.78, 0.88)	0
Cold ischemia time >14 hours	0.58	(0.41, 0.83)	0

Controlled donation	1.03	(0.93, 1.15)	0.53
Blood pH <7.0	2.71	(0.89, 8.21)	0.08
Regional allocation	0.9	(0.84, 0.96)	0
National allocation	0.68	(0.62, 0.74)	0
Weight difference at transplant 45-70kg	1.16	(1.02, 1.32)	0.03

ABO = A, B, O blood group system; BMI = body mass index; MELD = model for end-stage liver disease; INR = international normalized ratio; ICU = intensive care unit; OPTN = organ procurement and transplantation network; TIPS = transjugular intrahepatic portosystemic shunt.

Table S6: Prolonged length-of-stay multivariable logistic regression

**Recipient Factors**	**OR**	**95% CI**	***p*-value**
ABO compatible transplant	1	(0.88, 1.14)	0.952
Age >65	1.42	(1.28, 1.58)	0
Albumin 2.0-2.5 g/dL	1.07	(0.97, 1.19)	0.173
Ascites at transplant	1.18	(1.06, 1.30)	0.002
Bacterial peritonitis pretransplant	1.04	(0.93, 1.17)	0.452
BMI 30-35	0.94	(0.86, 1.02)	0.152
BMI 35-40	1.04	(0.93, 1.17)	0.495
BMI >40	0.98	(0.82, 1.16)	0.813
Creatinine 1.5-2.0 mg/dL	1.22	(1.09, 1.37)	0.001
Dialysis prior to transplant	1.65	(1.46, 1.87)	0
Education level - dropped out	1.13	(0.97, 1.31)	0.112
Education level - bachelor's degree	0.95	(0.87, 1.04)	0.298
Encephalopathy at transplant	1.47	(1.34, 1.62)	0
African American	1.14	(1.00, 1.30)	0.051
Hepatocellular carcinoma	0.87	(0.74, 1.01)	0.067
MELD 30-35	1.21	(1.06, 1.38)	0.005
MELD 35-40	1.27	(1.08, 1.49)	0.003
MELD >40	1.46	(1.19, 1.78)	0
Serum sodium 130-135 mEq/L	0.89	(0.81, 0.97)	0.006
Serum sodium 125-130 mEq/L	0.91	(0.81, 1.02)	0.114
Serum sodium 120-125 mEq/L	0.84	(0.69, 1.02)	0.083
Serum sodium 145-150 mEq/L	1.18	(0.97, 1.43)	0.102
Serum sodium 150-155 mEq/L	1.21	(0.84, 1.74)	0.31
Serum sodium 155-160 mEq/L	2.08	(0.91, 4.75)	0.083
Functional status at transplant: 2010	1.11	(0.94, 1.31)	0.216
Functional status at transplant: 2020	1.2	(1.07, 1.34)	0.001
Functional status at transplant: 2050	1.05	(0.92, 1.20)	0.449
Functional status at transplant: 2060	0.74	(0.64, 0.86)	0

Functional status at transplant: 2070	0.58	(0.50, 0.68)	0
Functional status at transplant: 2080	0.57	(0.47, 0.69)	0
Functional status at transplant: 2090	0.66	(0.50, 0.87)	0.003
Functional status at transplant: 2100	0.73	(0.44, 1.20)	0.213
INR 2.0-2.5	1.02	(0.92, 1.13)	0.72
INR 2.5-3.0	0.96	(0.85, 1.09)	0.555
INR 3.0-3.5	0.94	(0.80, 1.10)	0.438
INR 3.5-4.0	0.79	(0.63, 0.99)	0.041
INR > 4.0	0.81	(0.67, 0.98)	0.027
Life support pre-transplant	0.93	(0.80, 1.08)	0.318
ICU pre-transplant	1.95	(1.68, 2.27)	0
Hospital admission pre-transplant	1.48	(1.32, 1.67)	0
One previous transplant	1.32	(0.67, 2.59)	0.422
Two previous transplants	2.7	(1.48, 4.92)	0.001
Late re-transplant	1.31	(0.65, 2.65)	0.452
Early re-transplant	1.85	(0.91, 3.76)	0.089
Two late re-transplants	1	NA	0
Two early re-transplants	1.02	(0.29, 3.56)	0.979
Ventilator-dependent pre-transplant	2.11	(1.77, 2.52)	0
Portal vein thrombosis at registration	1.15	(0.99, 1.33)	0.072
Portal vein thrombosis at transplant	1.25	(1.11, 1.41)	0
Primary payment - private insurance	0.83	(0.76, 0.91)	0
Primary payment - Medicaid	1.02	(0.91, 1.15)	0.676
Previous abdominal surgery	1.1	(1.02, 1.18)	0.018
OPTN transplant region 1	1.13	(0.94, 1.36)	0.204
OPTN transplant region 2	1.36	(1.20, 1.55)	0
OPTN transplant region 3	0.86	(0.76, 0.97)	0.012
OPTN transplant region 5	1.03	(0.92, 1.16)	0.619
OPTN transplant region 6	0.87	(0.68, 1.10)	0.23
OPTN transplant region 9	1.46	(1.25, 1.71)	0
OPTN transplant region 10	0.96	(0.83, 1.11)	0.601
OPTN transplant region 11	0.8	(0.69, 0.92)	0.002
Total bilirubin < 2.0 mg/dL	0.92	(0.81, 1.04)	0.163
Total bilirubin 8.0-16.0 mg/dL	1.03	(0.91, 1.16)	0.601
Total bilirubin 16.0-32.0 mg/dL	0.95	(0.84, 1.08)	0.437
Total bilirubin > 32.0 mg/dL	1.21	(1.04, 1.41)	0.015
TIPS at transplant	1.18	(1.05, 1.33)	0.005
Working for income	0.65	(0.58, 0.72)	0
**Donor Factors**	**OR**	**95% CI**	***p*-value**

Donor age 15-20	0.94	(0.82, 1.07)	0.362
Donor age 20-30	0.88	(0.81, 0.97)	0.008
Donor age >70	1.07	(0.87, 1.32)	0.534
Cold ischemia time 0-6 hours	0.82	(0.76, 0.89)	0
Cold ischemia time >14 hours	0.87	(0.58, 1.29)	0.487
Controlled donation	1.06	(0.91, 1.24)	0.433
Distance from donor hospital 500-1000 mi	1.22	(1.02, 1.44)	0.027
Donor positive hepatitis C serology	1.01	(0.88, 1.17)	0.844
ALT/SGPT > 100 U/L	0.95	(0.87, 1.05)	0.312
Regional allocation	1.01	(0.92, 1.10)	0.864
National allocation	0.97	(0.87, 1.07)	0.535
Donor total bilirubin 1.0-1.8 mg/dL	1.12	(1.03, 1.23)	0.012
Weight difference at transplant 45-70kg	1.18	(1.00, 1.40)	0.056
Weight difference at transplant >70kg	1.32	(0.95, 1.85)	0.103

ABO = A, B, O blood group system; BMI = body mass index; MELD = model for end-stage liver disease; INR = international normalized ratio; ICU = intensive care unit; OPTN = organ procurement and transplantation network; TIPS = transjugular intrahepatic portosystemic shunt; ALT/SGPT = alanine aminotransferase/serum glutamic-pyruvic transaminase.

Table S7: 1-year graft failure multivariable logistic regression

**Recipient Factors**	**OR**	**95% CI**	***p-value***
ABO compatible transplant	1.14	(1.00, 1.31)	0.05
Age >65	1.41	(1.28, 1.55)	0
Albumin 2.0-2.5 g/dL	1.1	(1.00, 1.22)	0.056
Ascites at transplant	1.06	(0.97, 1.17)	0.179
BMI 35-40	1.17	(1.05, 1.31)	0.005
BMI >40	1.21	(1.03, 1.43)	0.023
Dialysis prior to transplant	1.32	(1.15, 1.51)	0
Encephalopathy at transplant	1.12	(1.01, 1.24)	0.031
African American	1.26	(1.12, 1.43)	0
Hepatocellular carcinoma	1.13	(1.01, 1.26)	0.04
MELD 35-40	0.8	(0.69, 0.92)	0.002
MELD >40	0.83	(0.70, 0.99)	0.044
Serum sodium 130-135 mEq/L	0.93	(0.86, 1.02)	0.119
Serum sodium 125-130 mEq/L	0.98	(0.87, 1.11)	0.744
Serum sodium 145-150 mEq/L	1.36	(1.12, 1.66)	0.002
Serum sodium 150-155 mEq/L	1.51	(1.05, 2.18)	0.028
Serum sodium 155-160 mEq/L	2.85	(1.40, 5.79)	0.004

Functional status at transplant: 2010	1.52	(1.27, 1.82)	0
Functional status at transplant: 2020	1.33	(1.18, 1.50)	0
Functional status at transplant: 2060	0.82	(0.73, 0.93)	0.003
Functional status at transplant: 2070	0.82	(0.73, 0.93)	0.001
Functional status at transplant: 2080	0.79	(0.69, 0.91)	0.001
Functional status at transplant: 2090	0.73	(0.58, 0.93)	0.01
INR >4.0	1.06	(0.87, 1.28)	0.56
Life support pre-transplant	1.09	(0.91, 1.30)	0.365
ICU pre-transplant	1.18	(1.03, 1.35)	0.02
One previous transplant	1.41	(0.72, 2.76)	0.309
Two previous transplants	4.4	(2.70, 7.16)	0
Late re-transplant	1.47	(0.74, 2.95)	0.275
Early re-transplant	1.38	(0.69, 2.79)	0.364
Two late re-transplants	1	NA	0
Two early re-transplants	0.39	(0.12, 1.19)	0.098
Ventilator-dependent pre-transplant	1.33	(1.08, 1.63)	0.007
Blood pH <7.0	3.87	(1.20, 12.49)	0.024
Portal vein thrombosis at registration	0.99	(0.86, 1.14)	0.904
Portal vein thrombosis at transplant	1.36	(1.21, 1.52)	0
Primary payment - private insurance	0.95	(0.87, 1.04)	0.258
Primary payment - Medicaid	0.89	(0.79, 1.00)	0.055
Previous abdominal surgery	1.15	(1.07, 1.24)	0
OPTN transplant region 1	1.22	(1.03, 1.46)	0.021
OPTN transplant region 2	1.22	(1.09, 1.37)	0.001
OPTN transplant region 6	0.91	(0.73, 1.15)	0.446
Total bilirubin 16.0-32.0 mg/dL	0.99	(0.88, 1.12)	0.878
Total bilirubin > 32.0 mg/dL	1.12	(0.95, 1.31)	0.18
TIPS at transplant	1.16	(1.04, 1.30)	0.01
Working for income	0.87	(0.79, 0.96)	0.008
**Donor Factors**	**OR**	**95% CI**	***p*-value**
Donor age <10	1.5	(0.98, 2.28)	0.061
Donor age 20-30	0.88	(0.80, 0.97)	0.009
Donor age 65-70	1.26	(1.13, 1.41)	0
Donor age >70	1.55	(1.30, 1.84)	0
Cold ischemia time 0-6 hours	0.79	(0.74, 0.85)	0
Controlled donation	1.68	(1.49, 1.89)	0
Distance from donor hospital 500-1000 mi	1.16	(0.99, 1.36)	0.075
Donor African American	1.15	(1.05, 1.26)	0.003

Donor Positive Hepatitis C Serology	0.94	(0.82, 1.09)	0.424
Height difference at transplant 30-60 cm	1.22	(0.94, 1.57)	0.129
ALT/SGPT > 100 U/L	0.94	(0.86, 1.03)	0.213
Regional allocation	0.97	(0.89, 1.05)	0.433
Weight difference at transplant 45-70kg	1.08	(0.91, 1.28)	0.366
Weight difference at transplant >70kg	1.27	(0.91, 1.78)	0.164

ABO = A, B, O blood group system; BMI = body mass index; MELD = model for end-stage liver disease; INR = international normalized ratio; ICU = intensive care unit; OPTN = organ procurement and transplantation network; TIPS = transjugular intrahepatic portosystemic shunt; ALT/SGPT = alanine aminotransferase/serum glutamic-pyruvic transaminase.

Table S8: DeLong's test for SOFT Cluster, BAR, SOFT, and MELD Scores

90-day mortality	AUC	95% Confidence Interval	Standard Error	*p*-value*
SOFT Cluster Score	0.6687	(0.657, 0.680)	0.0060	N/A
BAR Score	0.6059	(0.593, 0.618)	0.0063	0.000
SOFT Score	0.6510	(0.639, 0.663)	0.0061	0.002
MELD Score	0.5787	(0.566, 0.591)	0.0063	0.000
1-year mortality	AUC	95% Confidence Interval	Standard Error	*p*-value*
SOFT Cluster Score	0.6473	(0.638, 0.656)	0.0046	N/A
BAR Score	0.5830	(0.573, 0.592)	0.0048	0.000
SOFT Score	0.6262	(0.617, 0.635)	0.0047	0.000
MELD Score	0.5545	(0.545, 0.564)	0.0048	0.000
3-year mortality	AUC	95% Confidence Interval	Standard Error	*p*-value*
SOFT Cluster Score	0.643	(0.636, 0.650)	0.0036	N/A
BAR Score	0.5421	(0.534, 0.550)	0.0039	0.000
SOFT Score	0.5854	(0.578, 0.593)	0.0038	0.000
MELD Score	0.5156	(0.508, 0.523)	0.0039	0.000
5-year mortality	AUC	95% Confidence Interval	Standard Error	*p*-value*
SOFT Cluster Score	0.6384	(0.632, 0.645)	0.0032	N/A
BAR Score	0.5269	(0.520, 0.534)	0.0036	0.000

SOFT Score	0.5674	(0.561, 0.574)	0.0035	0.000
MELD Score	0.5010	(0.494, 0.508)	0.0035	0.000
Length-of-stay over 30 days	AUC	95% Confidence Interval	Standard Error	*p*-value*
SOFT Cluster Score	0.7550	(0.748, 0.762)	0.0035	N/A
BAR Score	0.7020	(0.695, 0.709)	0.0038	0.000
SOFT Score	0.7203	(0.713, 0.728)	0.0037	0.000
MELD Score	0.6886	(0.681, 0.696)	0.0038	0.000
1-year graft failure	AUC	95% Confidence Interval	Standard Error	*p*-value*
SOFT Cluster Score	0.6241	(0.616, 0.632)	0.0042	N/A
BAR Score	0.5574	(0.549, 0.566)	0.0043	0.000
SOFT Score	0.6020	(0.593, 0.610)	0.0042	0.000
MELD Score	0.5347	(0.526, 0.543)	0.0043	0.000

**p*-values derived from the DeLong's test compare each of the previously established scores (SOFT, BAR, MELD) to the SOFT Cluster score.

Table S9: Cox Regression and Harrell's *C*-statistic for SOFT Cluster, BAR, SOFT, and MELD Scores

1-year mortality	Hazard Ratio (95% CI)	*p*-value	Harrell's *C*-statistic (95% CI)
SOFT Cluster Score	1.129 (1.12, 1.14)	0.000	0.5165 (0.514, 0.519)
BAR Score	1.063 (1.06, 1.07)	0.000	0.4751 (0.472, 0.478)
SOFT Score	1.053 (1.05, 1.06)	0.000	0.4720 (0.469, 0.475)
MELD Score	1.018 (1.01, 1.02)	0.000	0.4751 (0.472, 0.478)
3-year mortality	Hazard Ratio (95% CI)	*p*-value	Harrell's *C*-statistic (95% CI)
SOFT Cluster Score	1.241 (1.23, 1.25)	0.000	0.5517 (0.549, 0.555)
BAR Score	1.036 (1.03, 1.04)	0.000	0.4734 (0.471, 0.476)
SOFT Score	1.039 (1.03, 1.04)	0.000	0.4726 (0.470, 0.475)
MELD Score	1.008 (1.00, 1.01)	0.000	0.4721 (0.469, 0.475)
5-year mortality	Hazard Ratio (95% CI)	*p*-value	Harrell's *C*-statistic (95% CI)
SOFT Cluster Score	1.398 (1.37, 1.43)	0.000	0.5790 (0.576, 0.582)
BAR Score	1.028 (1.02, 1.03)	0.000	0.4726 (0.470, 0.476)
SOFT Score	1.033 (1.03, 1.04)	0.000	0.4725 (0.470, 0.475)
MELD Score	1.005 (1.00, 1.01)	0.000	0.4708 (0.468, 0.474)

Table S10: Training and Validation Cohorts.

**Risk Factor**	**Number of Patients in Training Set**	**Percentage of Patients in Training Set**	**Number of Patients in Validation Set**	**Percentage of Patients in Validation Set**

Age 18-30	1605	3.93	818	4.01
Age 60-65	8411	20.60	4162	20.39
Age > 65	8755	21.45	4485	21.97
Albumin 2.0-2.5 g/dL	5640	13.82	2834	13.89
Ascites	30517	74.76	15433	75.61
BMI 35-40	4745	11.62	2321	11.37
BMI >40	2182	5.35	1149	5.63
Positive Hepatitis C Serology	4353	10.66	2138	10.48
Dialysis prior to transplant	5216	12.78	2610	12.79
Encephalopathy at transplant	5461	13.38	2754	13.49
Education level – high school	15696	38.45	7997	39.18
Education level – technical school	10365	25.39	5124	25.11
African American	3092	7.57	1542	7.56
Hepatocellular carcinoma	6657	16.31	3297	16.15
MELD 30-35	5325	13.04	2613	12.80
MELD 35-40	4082	10.00	2051	10.05
MELD >40	3704	9.07	1936	9.49
Serum sodium 130-135 mEq/L	11409	27.95	5721	28.03
Serum sodium 145-150 mEq/L	922	2.26	514	2.52
Serum sodium 150-155 mEq/L	211	0.52	76	0.37
Serum sodium 155-160 mEq/L	40	0.1	17	0.08
Moribund functional status (2010)	1952	4.78	1011	4.95
Very sick functional status (2020)	5841	14.31	2844	13.93
Requiring occasional assistance (2060)	5162	12.65	2603	12.75

Caring for self; unable to carry out normal activity (2070)	5957	14.59	2909	14.25
Normal activity with effort (2080)	4381	10.73	2169	10.63
Normal activity with minor symptoms (2090)	1453	3.56	741	3.63
INR 2.0-2.5	6766	16.57	3351	16.42
INR 3.5-4.0	884	2.17	457	2.24
INR > 4.0	1512	3.70	815	3.99
ICU pre-transplant	6207	15.21	3103	15.20
Hospital admission pre-transplant	8731	21.39	4438	21.74
One previous transplant	1512	3.70	762	3.73
Two previous transplants	111	0.27	40	0.20
Ventilator-dependent pre-transplant	1820	4.46	895	4.39
Portal vein thrombosis at transplant	5711	13.99	2836	13.90
Private insurance	21046	51.56	10503	51.46
Previous abdominal surgery	19557	47.91	9842	48.22
OPTN Region 1 – CT, ME, MA, NH, RI, East VT	1662	4.07	780	3.82
OPTN Region 2 – DE, DC, MD, NJ, PA, WV, North VA	4298	10.53	2233	10.94
OPTN Region 3 – AL, AR, FL, GA, LA, MS, PR	6921	16.95	3437	16.84

OPTN Region 5 – AZ, CA, NV, NM, UT	6301	15.44	3166	15.51
OPTN Region 6 – AK, HI, ID, MT, OR, WA	1300	3.18	606	2.97
OPTN Region 9 – NY, West VT	2343	5.74	1142	5.60
OPTN Region 11 – KY, NC, SC, OH, TN, VA	4103	10.05	2179	10.68
Total bilirubin <2.0 mg/dL	11724	28.72	5820	28.52
Total bilirubin >32.0 mg/dL	2738	6.71	1375	6.74
TIPS at transplant	4013	9.83	1995	9.77
Working for income	8458	20.72	4198	20.57
**Donor**				
Age 20-30	7845	19.22	3843	18.83
Age 65-70	4816	11.80	2450	12.00
Age >70	1587	3.89	751	3.68
Cold ischemia time 0-6 hours	22742	55.71	11334	55.53
Cold ischemia time 12-14 hours	463	1.13	223	1.09
Cold ischemia time >14 hours	433	1.06	258	1.26
Controlled donation	3746	9.18	1898	9.30
Creatinine 1.5-2.0 mg/dL	4082	10.00	2034	9.97
Distance from donor hospital 500-1000 mi	1804	4.42	878	4.30
African American	7378	18.07	3739	18.32
Positive Hepatitis C Serology	3435	8.41	1724	8.45
Blood pH < 7.0	16	0.04	12	0.06
Regional allocation	12155	29.78	6177	30.26

National allocation	6777	16.60	3358	16.45
Total bilirubin 1.0-1.8 mg/dL	7736	18.95	3701	18.13
Weight difference at transplant 45-70 kg	2012	4.93	1019	4.99
Weight difference at transplant -45 to -70 kg	1044	2.56	554	2.71
