# Supplementary material for: The SOFT Cluster Score as a Multifaceted Predictive Model for Postoperative Outcomes
Source: Transplant Direct. 2026 May 6;12(6):e1951. doi: 10.1097/TXD.0000000000001951 (PMC13155515; doi:10.1097/TXD.0000000000001951)
Supplement: Supplementary file 2 [file txd-12-e1951-s002.pdf]

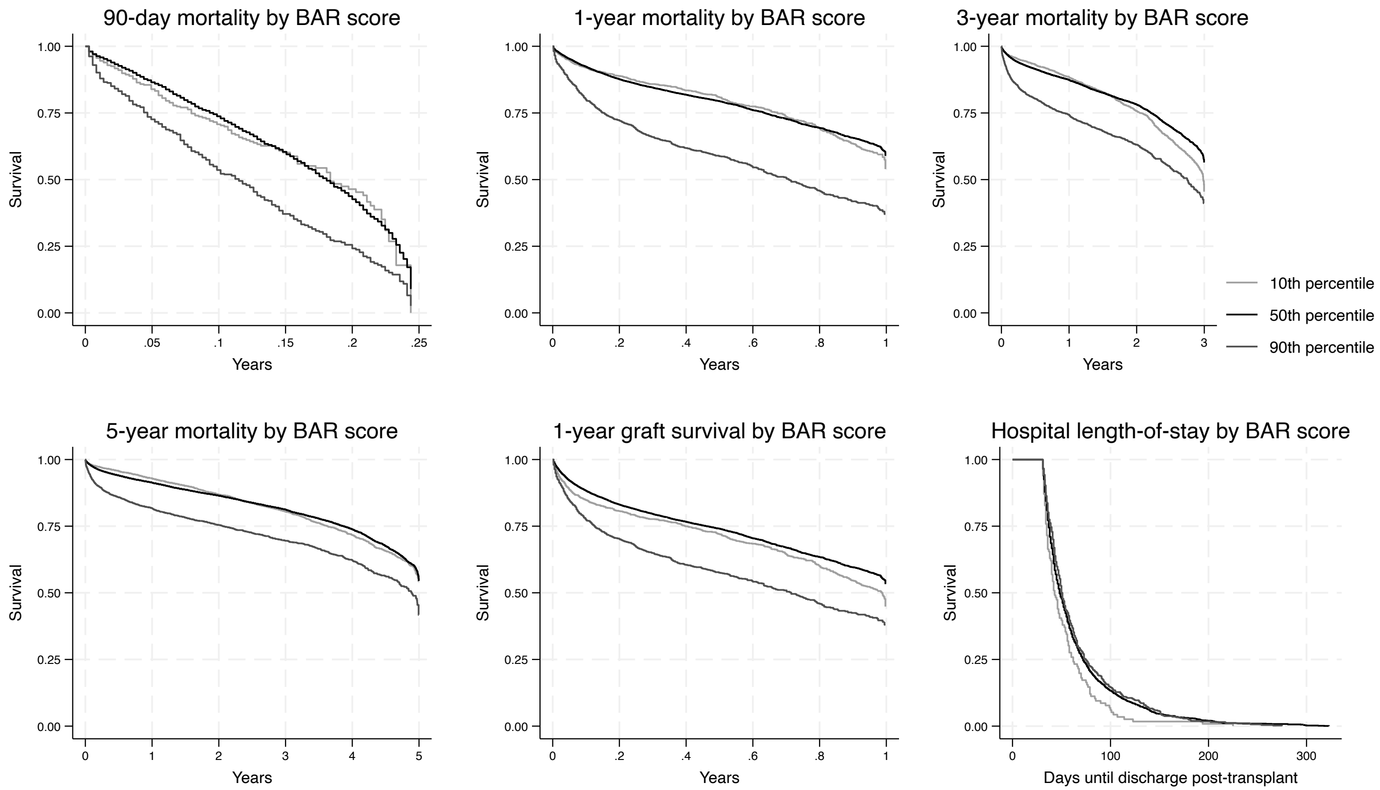
90-day mortality by BAR score
1-year mortality by BAR score
3-year mortality by BAR score
5-year mortality by BAR score
1-year graft survival by BAR score
Hospital length-of-stay by BAR score
Survival
Years
Days until discharge post-transplant
10th percentile
50th percentile
90th percentile
