# Supplementary material for: The SOFT Cluster Score as a Multifaceted Predictive Model for Postoperative Outcomes
Source: Transplant Direct. 2026 May 6;12(6):e1951. doi: 10.1097/TXD.0000000000001951 (PMC13155515; doi:10.1097/TXD.0000000000001951)
Supplement: Supplementary file 3 [file txd-12-e1951-s003.pdf]

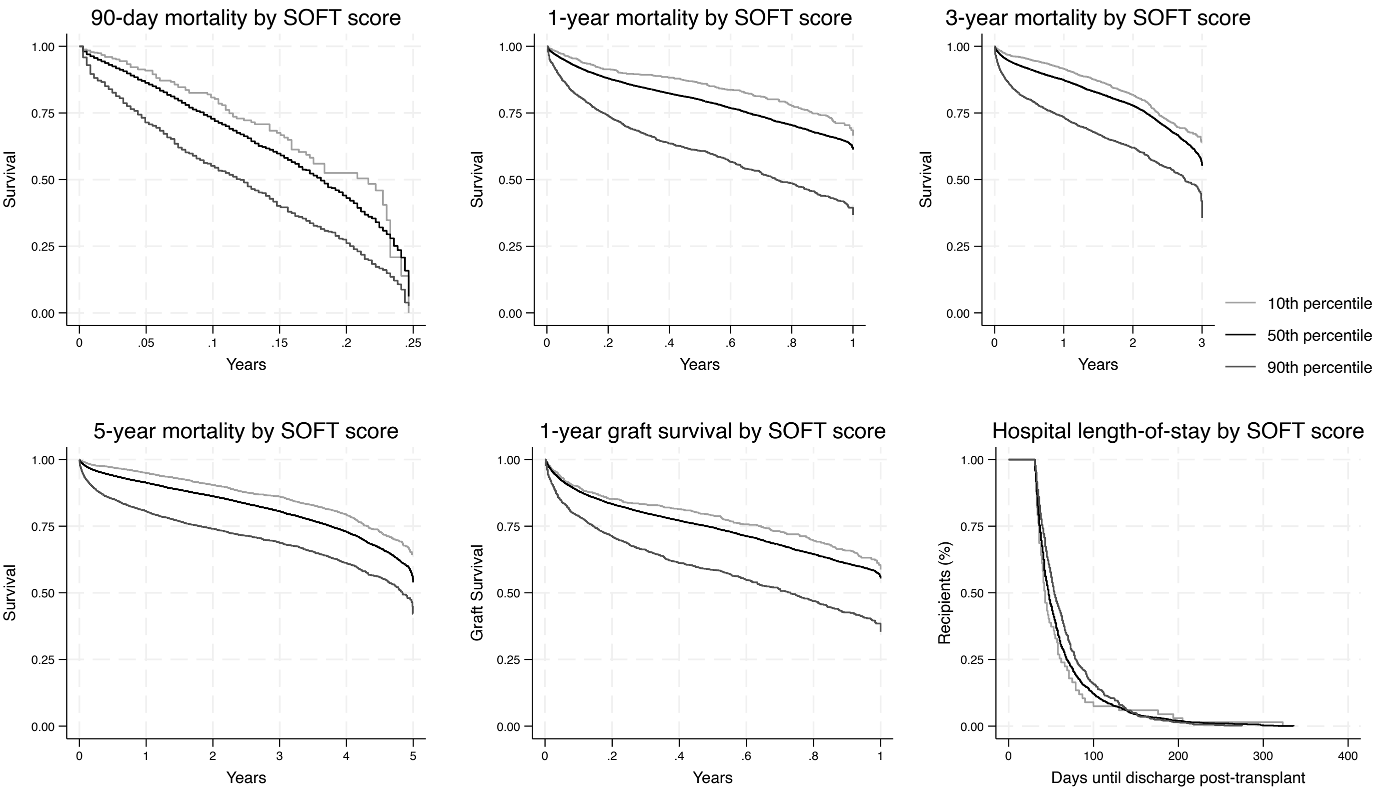
90-day mortality by SOFT score
1-year mortality by SOFT score
3-year mortality by SOFT score
5-year mortality by SOFT score
1-year graft survival by SOFT score
Hospital length-of-stay by SOFT score
Survival
Graft Survival
Recipients (%)
Years
Days until discharge post-transplant
1.00
0.75
0.50
0.25
0.00
0
.05
.1
.15
.2
.25
.4
.6
.8
1
2
3
4
5
100
200
300
400
10th percentile
50th percentile
90th percentile
