# Supplementary material for: The SOFT Cluster Score as a Multifaceted Predictive Model for Postoperative Outcomes
Source: Transplant Direct. 2026 May 6;12(6):e1951. doi: 10.1097/TXD.0000000000001951 (PMC13155515; doi:10.1097/TXD.0000000000001951)
Supplement: Supplementary file 4 [file txd-12-e1951-s004.pdf]

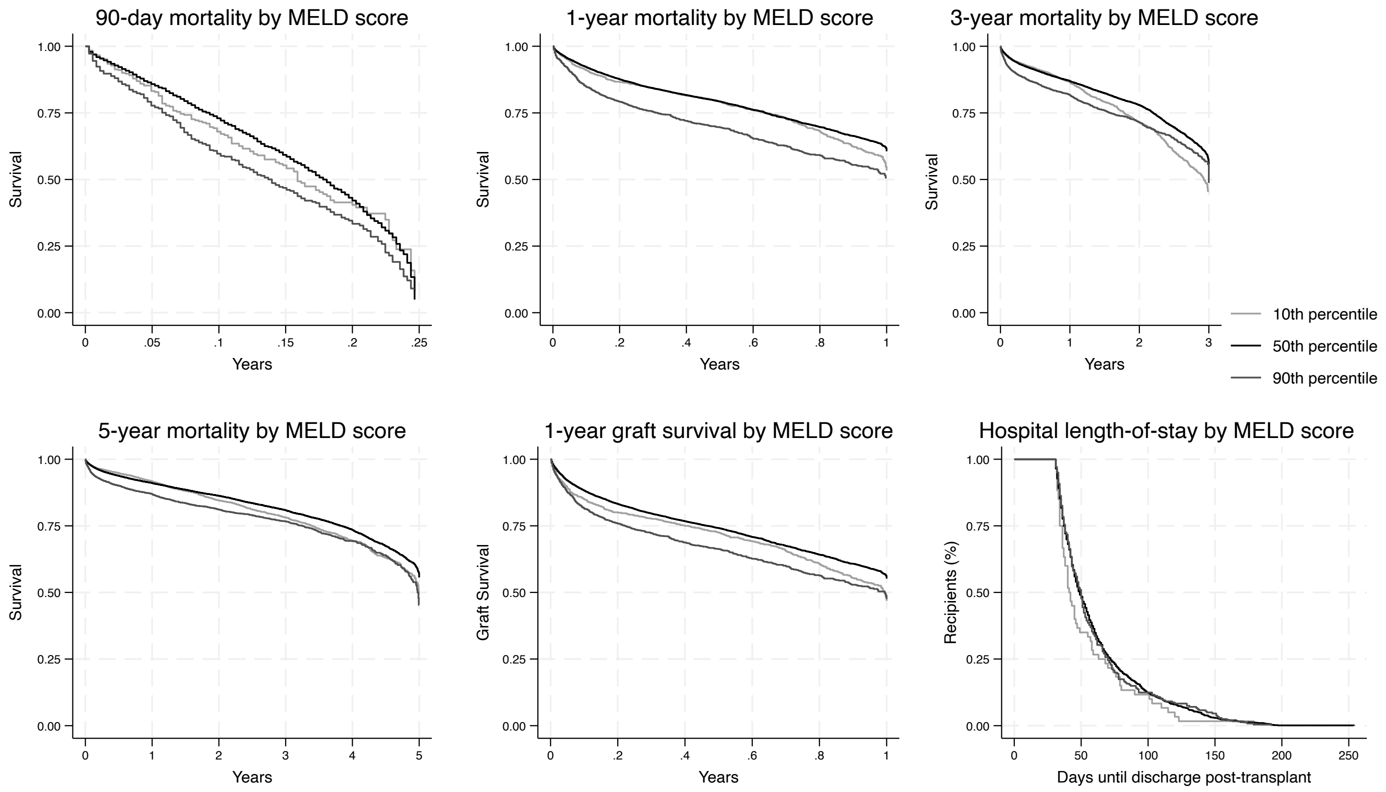
90-day mortality by MELD score
1-year mortality by MELD score
3-year mortality by MELD score
5-year mortality by MELD score
1-year graft survival by MELD score
Hospital length-of-stay by MELD score
Survival
Graft Survival
Recipients (%)
Years
Days until discharge post-transplant
1.00
0.75
0.50
0.25
0.00
0
.05
.1
.15
.2
.25
.4
.6
.8
1
2
3
4
5
50
100
150
200
250
10th percentile
50th percentile
90th percentile
